# Quantitative Analysis of the Interactions of Metal Complexes and Amphiphilic Systems: Calorimetric, Spectroscopic and Theoretical Aspects

**DOI:** 10.3390/biom12030408

**Published:** 2022-03-07

**Authors:** Rossella Migliore, Tarita Biver, Giampaolo Barone, Carmelo Sgarlata

**Affiliations:** 1 Institute of Biomolecular Chemistry, National Research Council, Via Paolo Gaifami 18, 95126 Catania, Italy; rossella.migliore@unict.it; 2 Department of Chemistry and Industrial Chemistry, University of Pisa, Via G. Moruzzi 13, 56124 Pisa, Italy; tarita.biver@unipi.it; 3 Department of Biological, Chemical and Pharmaceutical Sciences and Technologies, University of Palermo, Viale delle Scienze, Edificio 17, 90128 Palermo, Italy; giampaolo.barone@unipa.it; 4 Department of Chemical Sciences, University of Catania, Viale Andrea Doria 6, 95125 Catania, Italy

**Keywords:** metal complexes, amphiphilic systems, drug delivery, biological membrane, solution thermodynamics, speciation, isothermal titration calorimetry, spectrophotometry, molecular dynamics simulations, density functional theory calculations

## Abstract

Metals and metal-based compounds have many implications in biological systems. They are involved in cellular functions, employed in the formation of metal-based drugs and present as pollutants in aqueous systems, with toxic effects for living organisms. Amphiphilic molecules also play important roles in the above bio-related fields as models of membranes, nanocarriers for drug delivery and bioremediating agents. Despite the interest in complex systems involving both metal species and surfactant aggregates, there is still insufficient knowledge regarding the quantitative aspects at the basis of their binding interactions, which are crucial for extensive comprehension of their behavior in solution. Only a few papers have reported quantitative analyses of the thermodynamic, kinetic, speciation and binding features of metal-based compounds and amphiphilic aggregates, and no literature review has yet addressed the quantitative study of these complexes. Here, we summarize and critically discuss the recent contributions to the quantitative investigation of the interactions of metal-based systems with assemblies made of amphiphilic molecules by calorimetric, spectrophotometric and computational techniques, emphasizing the unique picture and parameters that such an analytical approach may provide, to support a deep understanding and beneficial use of these systems for several applications.

## 1. Introduction

Metals play essential roles in life processes since they are involved in cellular and subcellular functions. 

Potassium and sodium ions are the most abundant cations in the human body [1]. Sodium is at a higher concentration in the extracellular media of mammal organisms, while potassium exhibits a comparable concentration inside the cells. Other ions in the alkali metal series, such as lithium, rubidium and cesium, are present only at micromolar concentrations in the human body but are of medical/physiological relevance due to their curative or toxic properties depending on the concentration [2]. The interest in understanding the effect of metal ions on biological systems has attracted countless studies in this field for a long time. Cations such as potassium and sodium, which significantly reduce the electrostatic repulsion forces between the nucleotide phosphate groups that are negatively charged, allow the structure of the double-helix of DNA to be maintained and are crucial in determining the fine details of the helix geometry.

Divalent cations also play fundamental roles in biological processes. For example, Ca^2+^ or Mg^2+^ participate in the assembly of hard structures via bio-mineralization. Divalent metal ions are essential components of DNA polymerases, both for base excision and for catalysis of the transfer reaction [3], and stabilize histone–DNA interactions within nucleosomes [4].

Some metals, such as Cu, Fe and Zn, which are termed essential elements, have structural roles and are responsible for the stability of important biological molecules. In particular, they act as cofactors for enzymes involved in metabolism and cell growth and donate or accept electrons in redox reactions that are of primary importance in the generation and utilization of metabolic energy [5]. The specific mechanism of action of each metal depends on its physico-chemical and coordination properties. An excessive or reduced concentration of these elements, compared to that needed for biological functions, can be toxic for the organism.

Other metal ions are known mostly for their toxicity. Arsenic, cadmium, chromium, copper, lead, inorganic mercury, nickel, selenium and zinc are the pollutants listed by the United States Environmental Protection Agency (USEPA) as metals and metalloids commonly causing toxic effects [6,7,8]. The presence of these metals in the aqueous system leads to long-lasting dangerous effects for plants, aquatic life, humans, micro-organisms and animals. The metallurgy, pharmaceutical, leather, pesticide and fertilizer industries are the leading cause and sources of heavy-metal contamination. For example, the toxic effects of Hg, which exists in several forms in the environment, including elemental, organic and inorganic mercury, are well-known and mainly exerted on the nervous and excretory systems but also on the cardiovascular system [9,10,11]. In general, the accumulation of heavy metals in the human body results in severe injury to various organs, specifically the respiratory, nervous and reproductive systems and digestive tract [12,13].

Besides the significant impacts and roles played by the metal ions in living organisms, the use of metal-containing drugs to treat patients affected by different pathologies is well-established for their valuable therapeutic effect [14]. The incorporation of metals increases the activity of bioactive ligands and significantly enhances the pharmacological potential of inactive ligands. For instance, cisplatin and its derivatives (carboplatin, oxaliplatin, etc.) are currently the most widely used chemotherapeutic drugs [15]. Ruthenium-based compounds are promising candidates for their activity against specific cancer cell-types and low toxicity [16,17]. Intense research efforts have involved coinage complexes due to their cytotoxic effects on several cancers and their different pharmacological profiles compared to Pt-drugs [18,19].

More recently, suitably designed metal-based nanoparticles have found biomedical use for the treatment of infectious diseases [20]. Metal-based nanoparticles are the most prevalent inorganic nanoparticles due to their unique properties and multifaceted application potential. They are characterized by small sizes (10–100 nm) and can interact with biomolecules as well as within and on the cell surface; furthermore, their large surface area promotes cell permeability [21].

A significant obstacle that has limited the assessment and use of both metallodrugs and metal-based nanoparticles is their potential toxicity. In the latter case, the targeted drug delivery capability and therapeutic efficacy have been improved by conjugating selected ligands, proteins, antibodies and enzymes, which have a specific binding activity, to selected target cells [22]. In general, a strategy to decrease the toxicity, increase the bioavailability and improve the selectivity toward target cells is to employ proper nano-carriers and containers for drug delivery.

The use of drug-delivery systems based on micellar/vesicular aggregates represents an effective way of carrying drugs to their targets. Surfactants possess unique properties since their hydrophobic tails can assemble and form colloidal-sized particles in aqueous solutions above a certain concentration, the so-called critical aggregation concentration. Due to the hydrophobic environment of the core of these nano-aggregates (micelles, vesicles, liposomes, etc.), water-insoluble drugs can be easily solubilized and thus loaded for transport and targeted release [23]. 

The complexity of natural membranes requires models for their investigation at the molecular level, and the most commonly used membrane-mimicking systems are aqueous solutions of surfactants able to self-aggregate in micelles or vesicles. Surfactants have been employed in the removal of heavy metals by using different strategies such as soil-washing/extraction/desorption and phytoremediation [24]. Ultra-filtration methods, which take advantage of membrane pretreatment with surfactants/biosurfactants, have been successfully proposed for metal ion removal [25].

Since metal ions have multiple implications for biological systems, ranging from their involvement in cellular functions to their presence as pollutants in aqueous environments, and surfactant-based aggregates play a key role as models of biological membranes, nanocarriers, bioremediating agents, etc., an in-depth comprehension of the quantitative aspects related to metal ion–amphiphilic system interactions is crucial to understand the mechanisms and driving forces of the binding/recognition equilibria. However, despite a significant number of efforts to understand the metals or metal complexes partitioning micellar systems, the primary focus has remained of a qualitative nature. There is a general lack of quantitative data and thorough examination/discussion of such interactions and binding features, especially in terms of the thermodynamic stability, energetics of the reaction, equilibria in aqueous solution and speciation in biological fluids.

In this review, we summarize the relevant contributions in the literature to the quantitative investigation of the interactions of biologically relevant metal-based systems with assemblies made of amphiphilic molecules by analyzing results obtained by calorimetric, spectroscopic and computational techniques, to outline the current research on this interesting but not yet extensively explored topic.

## 2. Calorimetric Investigation of the Interactions of Metal-Based Compounds with Amphiphilic Systems 

Calorimetry has evolved significantly since its early beginning, and with its most recent technological evolution, has become a unique technique for monitoring chemical or biological events due to both its high sensitivity and the evidence that almost all chemical processes are associated with adsorption (or release) of heat [26]. One of the most common calorimetric techniques is titration calorimetry, in which the system’s composition is changed by adding one or more components and measuring the heat exchange. These experiments are typically run under isothermal conditions, and thus the technique is usually referred to as isothermal titration calorimetry (ITC). The main advantage of ITC is providing direct access to all the information needed to elucidate the thermodynamic profile of binding or dissociation reactions in solution, thus allowing us to obtain both the equilibrium constant and enthalpy of reaction values [27].

While ITC has primarily been used to investigate the thermodynamics of interactions between biological macromolecules and small molecules [28,29] or host-guest complex formation [30,31,32], it is becoming increasingly common for measuring binding interactions between assemblies of amphiphilic molecules (micelles, vesicles, etc.) and small molecules, metal ions or inorganic nanoparticles [33,34,35].

### 2.1. Solution Thermodynamics of Drug-Delivery Systems

The interest in functional drug-delivery systems has promoted the development of proper amphiphilic-based aggregates to be used for effective drug administration. Although micelle-based carriers for drug delivery have been widely described, quantitative characterization of the solution thermodynamics of these systems has rarely been addressed.

Few authors have employed isothermal titration calorimetry to determine the thermodynamic profile of the interactions between drugs and micelles. Kishore’s and Banipal’s research groups have reported quantitative insights into drug-surfactant interactions, investigating non-steroidal anti-inflammatory drugs (naproxen [36] and diclofenac sodium [36,37,38]), antibiotics (neomycin and lincomycin) [36], biological molecules (thiamine hydrochloride [39] and l-tryptophan [37]) and the anticancer molecule 5-fluorouracil [40] in the presence of cationic (hexadecyltrimethylammonium bromide, tetradecyltrimethylammonium bromide), anionic (sodium dodecyl sulfate) and non-ionic (TritonX-100) commercial surfactants.

In a typical calorimetric experiment, an aqueous solution of the drug is titrated into a solution of the surfactant in a micellar form. Calorimetric data are usually analyzed by the so-called “one-site binding model” [41,42], in which, after the *i*th injection, the total heat content (*Q*) of the solution in the sample cell (with an active cell volume *V*_0_) at fractional saturation Θ is determined from

(1) $$Q=n\Theta M_{t}\Delta HV_{0}$$

where $M_{t}$ is the total concentration of the surfactant, *n* is the number of available binding/partitioning sites on the host molecule and ∆*H* is the molar heat of the drug partitioning. 

For an injection volume *dV_i_*, the heat released ∆*Q(i)* from *i*th injection is given by Equation (2)

(2) $$\Delta Q\left(i\right)=Q\left(i\right)+\frac{dV_{i}}{V_{0}}\;\left[\frac{Q\left(i\right)+Q\left(i-1\right)}{2}\right]-Q\left(i-1\right)$$

A least-squares fit of the chosen model to the experimental data points allows for obtaining the binding/partitioning constant (K), enthalpy (∆*H*) and stoichiometry (*n*).

Quantitative characterization of the interactions between calixarene-based micelles and model drugs (antibiotics and anticancer agents) was also reported when analyzing data obtained by ITC measurements through a simplified model, which assumes the formation of a 1:1 species between the guest and the micellar aggregate [43,44]. The net heats of the reaction were analyzed by HypCal [45], software that allows for the simultaneous determination of the standard enthalpy and binding constant values, which has been specifically designed to treat data obtained from ITC instruments operating in the overfilled mode. The thermodynamic parameters are obtained by optimizing the agreement between observed and calculated reaction heats. Optimization is performed by non-linear least-squares analysis, minimizing the objective function (*U*):

(3) $$U=\sum^{\;}{\left(Q_{obs\;}-Q_{calc}\right)}^{2}$$

where *Q_obs_* is the observed heat for a given reaction step, corrected for dilution (blank) effects, while *Q_calc_* is calculated as:

(4) $$Q_{calc}=-\sum^{\;}\delta n\Delta H$$

where *δn* is the change in the number of moles of a reaction product and Δ*H* is the molar formation enthalpy of the reaction product. The sum is carried out over all the reaction steps; the squared residuals (*Q_obs_* − *Q_calc_*)^2^ are summed over all the titration points. Stability constant values and thermodynamic parameters are obtained by simultaneously analyzing calorimetric data obtained from different titrations.

A few papers described just the thermodynamic characterization of systems involving metal complexes and amphiphilic-based aggregates using calorimetry. Cheng et al. [46] combined low-molecular-weight fucoidan (LMWF_8775_, a sulfated polysaccharide), thermolysin-hydrolyzed protamine peptide (a cell-penetrating peptide, TPP_1880_) and Gd-DTPA (a magnetic resonance imaging, MRI, contrast agent) in a nanosystem for in vitro MR imaging of the inflammatory endothelial cell model. The authors employed nano-ITC to investigate the interaction between TPP_1880_ and LMWF_8775_, identifying two reaction stages attributed to the formation of TPP_1880_/LMWF_8775_ ion pairs and the complex coacervation of TPP_1880_/LMWF_8775_ ion pairs, respectively. Two association constant and enthalpy values were determined, indicating that the binding affinity of the TPP_1880_/LMWF_8775_ ion pair that formed at the first step was stronger than that for the second event. The *n* values were found to be 1.73 and 13.2 for the first and second reaction steps, respectively. The first value represents the stoichiometric TPP_1880_/LMWF_8775_ ratio required to form the TPP_1880_/LMWF_8775_ ion pair and indicates that TPP_1880_ binds to LMWF_8775_ in a non-specific mode (multiple binding sites). The stoichiometry number (*n*) of the complex coacervation of TPP_1880_/LMWF_8775_ ion pairs (the second reaction stage) is explained by assuming that the base groups in TPP_1880_ were shielded during the process of self-assembly into the complex coacervates. 

**Figure 1:**
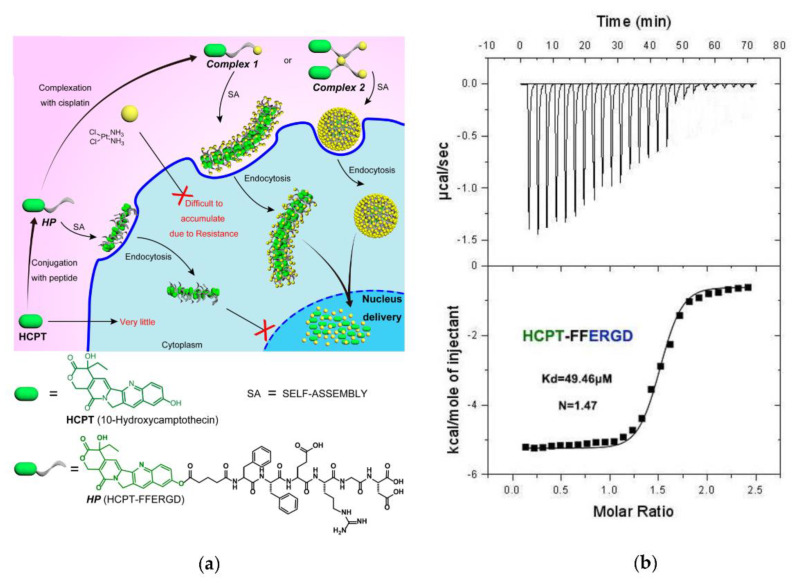
(**a**) Schematic illustration of the preparation of dual-drug assemblies and nuclear drug delivery (above); chemical structures of HCPT and HP (below). (**b**) ITC titration curves of HP with cisplatin. Figure adapted with permission from reference [47]. Copyright 2017 American Chemical Society.

Cai et al. [47] reported on a drug-delivery system in which the drug is amphiphilic itself, i.e., it is able to spontaneously self-assemble into nano-aggregates that serve as both carriers and cargos. They choose 10-hydroxycamptothecine (HCPT) and cisplatin to develop a dual anticancer drug assembly by synthetizing a HCPT-peptide (HP) amphiphile (Figure 1a). Cisplatin was chosen since it is able to form complexes with biopolymers and peptides containing carboxylic acid groups [48]. In their pioneering work, before examining the effect both in vitro and in vivo of the synthesized supramolecular nanostructure, the authors investigated the interaction between the amphiphilic drug HP and cisplatin through ITC by titrating a cisplatin solution into the peptide solution. The calorimetric data and the binding plot were analyzed using the one-site binding model, finding that HP was able to bind and suggesting that the two anticancer drugs could be taken up simultaneously by cells (Figure 1b).

In general terms, although the interactions and binding processes involved in these multi-component micellar-based systems are intrinsically complex, making quantitative characterization hard to be accomplished, a thorough understanding of these phenomena may be obtained from the analysis of thermodynamic data from isothermal titration calorimetry, which provides key parameters for assessing the binding events and driving forces of the molecular recognition equilibria in solution by directly measuring the heat of the reaction. 

### 2.2. Metal Ions’ Binding with Model Membranes

The interaction of metal ions with membranes has attracted significant interest in recent decades, mostly focusing on characterizing ion binding to lipid bilayers and on the corresponding change in the membrane surface potential [49,50,51]. Indeed, micelles and vesicles have become a topic of great interest to chemists and biochemists due to their similarity to biological membranes and globular proteins. 

The first systematic study dealing with the binding equilibria of alkali metal chlorides with lipid vesicles, studying palmitoyloleoylphosphatidylcholine (POPC) at physiological salt concentrations, was reported by Dimova’s group in 2010 [52]. The ITC measurements were carried out by titrating a solution of POPC large unilamellar vesicles (LUVs) with alkali metal chloride solutions. The calorimetric data allowed the group to determine the binding enthalpy by using Equation (5):

(5) $$\delta q=\delta n_{lip}^{b}\Delta H$$

where $\delta n_{lip}^{b}$ is the change in the moles of lipid bound by the cations after the injection. Assuming a simple partition equilibrium of 1:1 ion-to-lipid complex formation, the apparent binding constant *K* was defined as

(6) $$K\approx \frac{C_{lip}^{b}}{C_{ion}\left(C_{lip}-C_{lip}^{b}\right)}$$

where $C_{lip}$ is the total concentration of lipids available for binding and the difference $C_{lip}-C_{lip}^{b}\;$is the concentration of unbound lipids. The authors then assumed that the concentration of the unbound ions was approximately equal to the total ion concentration $C_{ion}$. Combining the two equations and inserting the derivative with respect to the total electrolyte concentration $C_{ion}$, they obtained Equation (7):

(7) $$\delta q=\delta C_{ion}\frac{KC_{lip}}{{\left(1+KC_{ion}\right)}^{2}}V_{cell}\Delta H$$

Fitting the data through Equation (7), they obtained the apparent binding constant *K* and the reaction molar enthalpy Δ*H*. The thermodynamic parameters showed that the binding of ions to phosphocholine bilayers is an entropically driven process, but enthalpically unfavored. The gain in entropy was attributed to the release of several water molecules from the hydration shell of the ions and the dehydration of the lipid membrane. The authors also observed that the (endothermic) reaction enthalpies increase in absolute value when the size of the bare cation decreases, following the Hofmeister series. 

Maity et al. [53] investigated the affinity of various monovalent alkali cations with the negatively charged dioleoylphosphatidylcholine (DOPC) and dioleoylphosphatidylglycerol (DOPG) molecules used for vesicles’ preparation. ITC data were analyzed by using the partition model, and the binding heats for all alkali metal ions to vesicles were evaluated. Comparison of the results with some reference measurements revealed that, besides ion binding, other forces were contributing to the ion–membrane interaction process. The observed entropy gain was attributed to the release of water molecules from the hydration shell of the ions and the hydration layer in the surroundings of the membranes. This work emphasized the significance of determining thermodynamic parameters contributing to the free Gibbs energy since the association constant values alone cannot provide information and details on the forces driving a binding process.

Calorimetric studies on the interaction of metals of biological interest with model membranes have also been reported. Kerek et al. [54] examined the interaction of Hg^2+^ and Cd^2+^ with different lipid structures under physiological conditions to better understand the metal-induced effects on potential targets in biomembranes. Since Hg and Cd are toxic for the central nervous system [55,56], vesicles containing different phospholipids at different compositions were prepared to create biomimetic lipid models of the human myelin sheath. Several techniques were employed to analyze the influence of both metal ions on the membrane fluidity. The initial screening identified phosphatidylethanolamine plasmalogen (PEplasmas) as the only target for Hg-induced membrane rigidity. Phosphatidylcholine plasmalogen (PCplasm) was studied as well to compare two enol/ether lipids with different headgroup architectures (choline vs. ethanolamine). The metal-lipid interaction was investigated by ITC titrations. The interaction of Hg with both PC and PE plasmalogens was found to be enthalpically favored, with a more favorable entropic contribution in the case of PEplasm. Despite the identical vinyl ether bonds in both plasmalogens, the difference in the choline and ethanolamine headgroups was significant as Hg bound PEplasm more strongly than PCplasm, thus revealing specific metal-lipid interactions that may help to understand the toxicology of these systems.

Garidel et al. [57] studied the binding reaction of Mg^2+^, Ca^2+^ and Sr^2+^ with 1, 2-dimyristoyl-sn-glycero-3-phosphatidic acid (DMPA) vesicles, used as model membranes, to assess ion-specific lipid–divalent cation interactions. ITC experiments were conducted in the “batch” mode, where the DMPA vesicle suspension was injected into a highly concentrated aqueous solution of the metal ion, which ensured the presence of a large excess of M^2+^ throughout the experiment. The binding constants’ values were large enough that almost complete saturation of DMPA vesicles with the cations was achieved after addition into the solution containing excess divalent cations. This approach was employed since a titration of the divalent cation solution into a vesicle suspension would lead immediately to aggregation and precipitation of the vesicles, making it impossible to record a complete titration curve, whereas in the “batch” mode, the total reaction enthalpy was measured. The calorimetric experiments were performed in the temperature range between 8 and 70 °C, which allowed the change in the heat capacity (Δ*C*_p_) to be determined for the binding process. This parameter describes the hydration changes caused by electrostatic interactions at the bilayer–water interface.

The above cited works investigated the direct interactions between metal ions and membrane models. However, many processes of biological significance implicate the interaction between metals and proteins that are within biological membranes. In this case, one has to consider both the interaction between the metal ion and the protein and the role played by the biological membranes in the whole process.

In this framework, Li’s group explored the interactions between human copper transporters (hCtr1 and hCtr2) and silver ions; members of the Ctr class of copper transporters are crucial for mediating cellular import of silver, although the mechanisms involved in the absorption of silver into the body are poorly understood. Studies were carried out using ITC in the presence of sodium dodecyl sulphate (SDS) micelles as biomimetic membrane systems. In their first paper [58], the authors investigated a 20-residue peptide from the extracellular second methionine-rich motif (M2-motif) inclusive sequence of hCtr1 (the segment from Gly34 to Asn53) in the presence of AgNO_3_. Calorimetric experiments were carried out by titrating a peptide solubilized in an SDS micellar solution with AgNO_3_ (dissolved in an SDS micellar solution). The effect of substituting alanine with methionine in the peptide was also examined. As a comparison, titrations were also conducted by using aqueous solutions of the reagents only (without SDS micelles) to unveil the role played by the biological membrane. The thermodynamic parameters, obtained by fitting the experimental data using the one-site binding model, indicated that the binding of the micelle-bound peptide is different from that of the peptide in the solution state. The binding affinity of the peptide in SDS micelles was found to be larger than that in water. Furthermore, the association process of the micelle-bound peptide with Ag^+^ was less exothermic than that of the peptide in water, compensated by less negative entropy change, indicating that more hydrophobic interactions are involved in the complexation of a micelle-bound peptide with Ag^+^. The solution thermodynamics yielded by calorimetry clearly indicated that the interaction between the extracellular domain of hCtr1 and the membrane surface is fundamental in the binding structure and binding process of the peptide with the ligands.

**Figure 2:**
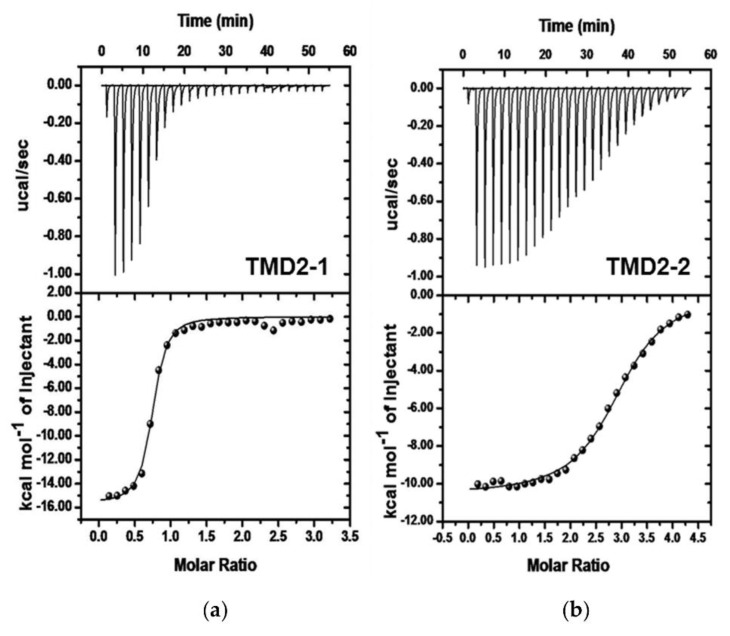
ITC profiles of (**a**) 50 μM peptides TMD2-1 and (**b**) TMD2-2 in 10 mM SDS micelles at pH 6 titrated with AgNO_3_. Figure adapted with permission from reference [59]. Copyright 2015 American Chemical Society.

Subsequently, they described the binding of the second transmembrane domain (TMD2) of hCtr1, hCtr1−TMD2, to Ag^+^ in SDS micelles used as membrane-mimicking agents [59]. ITC experiments (Figure 2) carried out in the presence of different forms of the peptide, in which methionines were converted to leucines, allowed the authors to gain insights into the roles of the methionine residues, both in the peptide assembling and in the binding of Ag^+^. The complexation processes were driven by enthalpy changes in the case of the peptides containing methionine residues. In contrast, the entropic contribution was found to drive the process in the absence of methionine, emphasizing the role of the peptide structure for the binding of the metal ion.

The same group also studied hCtr2 by analyzing the binding of TMD2 of hCtr2 to silver in the presence of SDS micelles using calorimetry; the results indicated that the reaction is exothermic and hydrogen-bonding and van der Waals interactions are the driving forces for the binding of the peptide to the silver ion [60].

More recently, Yang et al. [61] evaluated the binding features of the copper transport protein hCtr1 by reporting the effect of Cu^+^ in the interaction between hCtr1 and dipalmitoyl-phosphatidylcholine(DPPC) liposomes. Calorimetric experiments were carried out by titrating a solution of a peptide from hCtr1 (with or without Cu^+^ salts) into a liposome solution and data were fitted through a single-site binding model. Significant enthalpy changes occur only in the presence of Cu^+^, and this event is even more pronounced when two equivalent cuprous ions are added. The results revealed that Cu^+^ binding enhances the hCtr1 interaction with cell membranes.

Of note, in the reviewed papers, two approaches were employed to study the interactions of amphiphiles mimicking cellular membranes with selected peptides and metal ions. In one case, the micellar solution was used as a “medium” in which metal ions and peptides were dissolved to simulate the cellular environment [58,59,60]. In these experiments, the interactions measured were those occurring between the metal ion (titrant) and the peptide (titrate), while the effect of the micellar aggregate was determined by carrying out experiments both in the presence and absence of the amphiphilic system. In the other case, the interaction between the peptide and the surfactant-based aggregates (e.g., liposomes) was directly monitored by titrating a solution of liposomes with a peptide solution [61]. The effect of the metal ions was evaluated by conducting experiments in which the peptide solution either contained or did not contain metal ions. In our mind, both approaches are apt for investigating these rather complex systems; the choice may depend on which kind of interaction needs primary quantitative determination.

### 2.3. Micelles/Vesicles for Nanoparticles’ Stabilization

Binding processes also occur, and need to be quantitatively analyzed, when surfactants are employed in the stabilization of metal nanoparticles. Panda’s research group reported the synthesis and characterization of colloidal dispersions of silver nanoparticles in the presence of cationic surfactants that may work as a source of counterion (bromide o chloride) and as a stabilizing agent. They initially described the features of colloidal dispersions of silver bromide obtained by progressive addition of silver nitrate to an aqueous solution of hexadecyltrimethylammonium bromide (CTAB), which acted both as a stabilizer and as the source of bromide ions [62].

**Figure 3:**
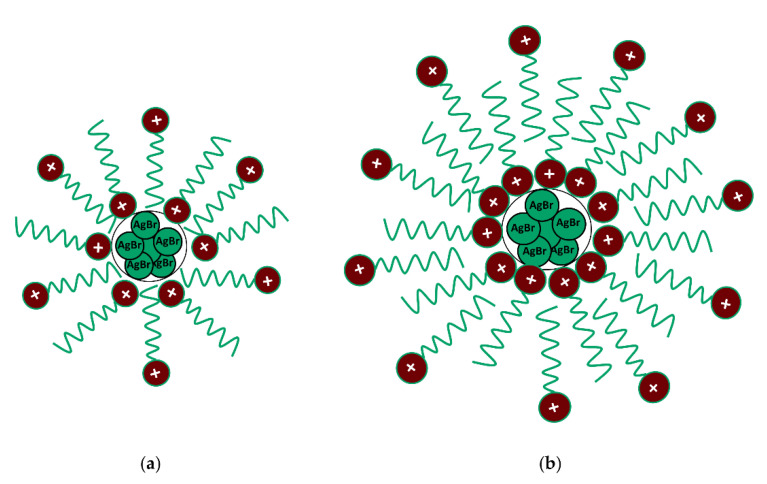
Schematic representation for (**a**) surfactant monolayer-protected AgBr nanoparticles and (**b**) AgBr nanoparticles stabilized by CTA^+^ bilayer. Figure adapted with permission from reference [62]. Copyright 2012 American Chemical Society.

Later, a nanocolloidal dispersion of silver chloride was synthesized in an aqueous medium using three cationic surfactants: hexadecyltrimethylammonium chloride (CTAC), hexadecylpyridinium chloride (CPC) and benzyldimethylhexadecylammonium chloride (BDHAC) as the source for the chloride ions and as the stabilizing agents [63]. The three cationic surfactants were used to further investigate the stability dependence of the synthesized silver chloride colloidal nanoparticles on the nature of the surfactant head group. The colloidal dispersions of silver nanoparticles were stabilized by the layered structured and positively charged surfactant assemblies (Figure 3). In these works, ITC calorimetry was employed to measure the enthalpy changes for the formation of nanoparticles, obtained by titrating a solution of silver nitrate into a cationic surfactant aqueous solution; the process was always found to be enthalpically favored.

Wang et al. [64] examined the binding of surfactants with different alkyl and head groups, i.e., sodium oleate (SO), sodium laurate (SL), sodium dodecyl sulfate (SDS) and sodium dodecylphosphonate (SDP), with iron oxide nanoparticles and evaluated the effects on the colloidal stability. Surfactants with longer alkyl tails displayed larger apparent binding enthalpies; different surfactant head groups also yielded varying affinities for iron oxide nanoparticles. The amphiphiles that displayed larger apparent binding enthalpies (endothermic or exothermic) were found to form stable suspensions of iron oxide nanoparticles, suggesting that large Δ*H* values are associated with a larger amount of surfactant bound to the surface of the particles. 

The binding curve obtained for the sodium oleate/iron oxide system was analyzed by using Freundlich, Langmuir and Langmuir–Freundlich isotherms, and the latter displaying the best fit due to the combination of the non-homogenous adsorption features of the Freundlich with the finite monolayer adsorption of the Langmuir equation.

## 3. Spectroscopic Analysis of Metal Complexes in Amphiphilic Media

Investigation of systems containing metal-ligand complexes in micellar/vesicular media may be conducted using plenty of different techniques, among which we may cite fluorescence [65] or atomic force microscopy [66]. However, since often at least the ligand possesses some absorbance in the UV-Vis range, simple spectrophotometry can play a crucial role in this examination, and spectrophotometric data can also be used to analyze the mechanism of complex formation in amphiphilic media, both from the thermodynamic and kinetic points of view. Sometimes, one of the species may emit light and a fluorescence signal may be recorded. Note that metal ions may be also directly coordinated to the surfactant head, leading to the so-called metallo-surfactants (or metal surfactant complexes), which are a recent alternative to conventional surfactants possessing unique properties, opening the door to different applications [67,68]; however, these topics are out of the focus of the present section devoted to metal-ligand equilibria in amphiphilic media. 

### 3.1. Interaction of Metal Ions with Lipid Membrane Mimicking Systems

**Figure 4:**
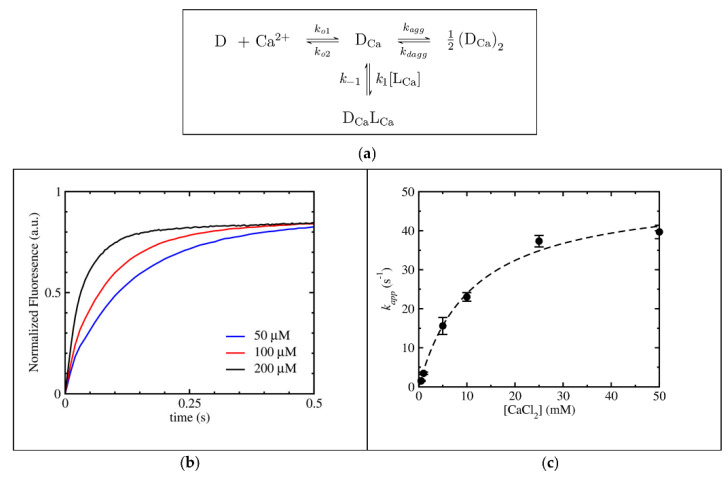
Kinetic analysis of the binding of daptomycin to lipid vesicles in the presence of calcium ions. (**a**) Kinetic model; (**b**) kinetics of daptomycin binding to lipid vesicles as a function of the lipid concentration; (**c**) apparent rate constant of binding, k_app_, dependence on the calcium ion concentration. Figure adapted with permission from reference [71]. Copyright 2018 American Chemical Society.

The presence of physiological metal ions may play an important role in modulating the efficacy of bio-active compounds. A system that was the object of intense study is daptomycin/Ca^2+^. Daptomycin is a cyclic polypeptide that has been in clinical use since 2003 to treat severe infections by Gram-positive drug-resistant bacteria. It was found that the activity of this important antibiotic depends critically on the presence of calcium ions. Recent work showed that the Ca^2+^-daptomycin complex interacts specifically with cell envelope precursors in the presence of an anionic phospholipid, forming a tripartite complex and explaining the specificity of daptomycin for bacterial cells [69]. Ca^2+^ is necessary for daptomycin-binding to the lipid membrane, mimicking cell walls that perturb the latter to a significant extent, causing the formation of domains and major reorganization essential to the antibiotic efficacy [70]. In a detailed kinetic study, the fluorescence resonance energy transfer (FRET) between the intrinsic Kyn residue of daptomycin and a fluorescent tag incorporated in the lipid membrane was used to monitor peptide-binding and dissociation from the vesicle-mimicking cell walls [71]. The kinetic model used to fit the data is shown in Figure 4a. Here, D is the daptomycin monomer, D_Ca_ is Ca^2+^-bound daptomycin, (D_Ca_)_2_ is a Ca^2+^-bound daptomycin dimer, D_Ca_L_Ca_ is the daptomycin monomer bound to a Ca^2+^-complexed lipid and L_Ca_ is the concentration of Ca^2+^-complexed lipid. The detailed kinetic study (using a stopped-flow technique with fluorescence detection) over different experimental conditions and reactant contents enabled the mechanistic (both equilibrium and kinetic) aspects of this complex system to be determined. On the whole, these experiments were able to describe the early events that occur in the binding of daptomycin to lipid bilayers and revealed the high affinity for the Ca^2+^-complexed lipid. 

Kinetic studies on the behavior of biologically relevant cations may also concern redox reactions. The reduction of Cr^6+^ to Cr^3+^ was examined in parallel with studies on the micellar effects on the lysis of organic systems [72,73]. Cr^6+^ is a known carcinogen, still present as a pollutant coming from industrial wastes. Its reduction occurs in the cell cytoplasm. The cited work [74] used micelles to mimic the cellular membranes as a model to gain information on the process occurring in the body. Spectrophotometric kinetic experiments were carried out by monitoring the decay of oxidant [Cr^6+^] at 415 nm at different time intervals. They discerned the details of the mechanism of the reduction reaction by 1-butanol in the presence of a cationic (*n*-cetyl pyridinium chloride, CPC), an anionic (sodium dodecylsulfate, SDS) or a neutral (TritonX-100) surfactant. In all cases, monomeric Cr^6+^ undergoes redox decomposition through two-electron transfer at the rate-determining step and a kinetic law of the first order on both reactants and the second order on the [H^+^] ion. The accelerating or decelerating effects of the surfactants were discussed on the basis of the partitioning of the reactants over different parts of the micellar phase (Gouy-Chapman or Stern layer).

### 3.2. Metal Complexes Speciation in Amphiphilic Media

Amphiphilic systems may profoundly alter the equilibria of a metal complex formation, thus producing speciation profiles that differ from those commonly known and applied in water. Researchers need to carefully take into account that both the species distribution and protonation profile on the micellar surface may change, in particular, when the amphiphilic system is bearing some charge. For instance, metallochromic indicators based on azo-ligands such as pyridine-2-azo-p-dimethylaniline (PADA) or 4-(2-pyridylazo)resorcinol (PAR) were used to tune the affinity for the micellar aggregate: the ligand is attracted by the hydrophobic core of the aggregate and drives the accumulation of the metal ion it coordinates. However, the ligand will have apparent acid dissociation constants, which differ from that in water. Indeed, PADA is a ligand with a p*K_A2_* (PADAH^+^ PADA + H^+^) of 4.5 ± 0.2 in aqueous solution, which is shifted to an apparent p*K_A2_* of 6.7 ± 0.2 in the presence of negatively charged micellar SDS; neutral PAR protonates with p*K_A1_* = 2.5 and p*K_A2_* = 5.5 in water and apparent p*K_A1_* = 4.0 and p*K_A2_* = 7.0 in SDS [75]. In the presence of positively charged micelles by dodecyltrimethylammonium chloride (DTAC), due to the opposite effect on the local concentration of H^+^ ions, the induced shift on the p*K_A_* is also reversed: for PADA p*K_A2_* = 2.6 in DTAC [76].

∆p*K_A_* = p*K_A_*(micelle) − p*K_A_*(water) (8)

Thus, the apparent p*K_A_* shift (Equation (8)) is mainly associated with the higher/lower local concentration of hydrogen ions at the micelle surface, which disfavors/favors the acid dissociation process of the dye, respectively. Owing to the difference in proton concentration, any comparison between data in a micellar media solution and water should be made at an “equivalent” hydrogen ion concentration using Equation (9) [77]

pH(micelle) = pH(measured) − ∆*pK_A_* (9)

Note that Equation (9) should be applied only when the system is such that the reaction totally occurs on the micelle surface. Finding the conditions where this occurs significantly simplifies the intricate analysis of these complex systems. Conversely, if this is not the case, the overall data/numbers evaluated will be the weighted sum of the contributions of water and micellar behaviors (see, for instance, Equation (13) below). Also, the discussion above on ∆*pK_A_* amplitude/sign considers how the charged micellar surface affects [H^+^]. However, different and even opposite effects on ∆*pK_A_* with respect to the electrostatic hypothesis may be found if the ligand is buried in the micellar core; this is particularly true when the ligand is highly hydrophobic [78]. 

The ∆*pK_A_* aspects may be analyzed more in the detail to obtain information on the surface potential experienced by the acid-base ligand and on its position on the surfactant aggregate. The relationship between proton activity on a surface (*a^H+^_S_*) of some potential *ψ* (mV) and that in the bulk water (*a^H+^_W_*) is

(10) $$\frac{a_{S}^{H+}}{a_{W}^{H+}}=e^{-\frac{F\psi }{RT}}\;\;\;\;\overset{\;\;\;\;\;\;T=25{}^{\circ}C\;\;\;\;\;\;\;}{\Rightarrow }\;\;\;\;pH_{S}-pH_{W}=\frac{\psi }{59.2}$$

where *F* is the Faraday constant, *R* the gas constant and *T* the temperature. Equation (10) was derived by Hartley and Roe on the basis of Boltzmann’s law of charged particle distribution in the presence of an electrically charged surface; note that *ψ* will have the same sign as the micelle charge [79]. Equation (10) can be further developed into Equation (11)

(11) $$pK_{A}^{S}-pK_{A}^{i}=-\frac{F\psi }{2.3RT}$$

where p*K_A_^i^* is used instead of p*K_A_^W^* to account also for any intrinsic effect arising from changes in the environment. Under the hypothesis that the contribution of the local environment does not change, regardless of the surface charge, we may state p*K_A_^i^* = p*K_A_*^0^ where p*K_A_*^0^ is the p*K_A_* of the probe measured in the presence of uncharged micelles (such as, for instance, Triton X). Given this theoretical basis, spectrophotometric acid-base titrations may be repeated in water or in the presence of differently charges species. This procedure enables the calculation of p*K_A_^S^* − p*K_A_*^0^ and thus of *ψ* in some charged micellar mediums. The comparison of the ψ value found with that of the inner potential (Ψ) and the zeta potential (ζ) of the micelle enables us to gain information on the positioning of the deprotonation sites of the acid-base ligand, which may be located either in the inner core or at the outer surface of the double layer, as well as protruding out of the surface toward the bulk water. 

**Figure 5:**
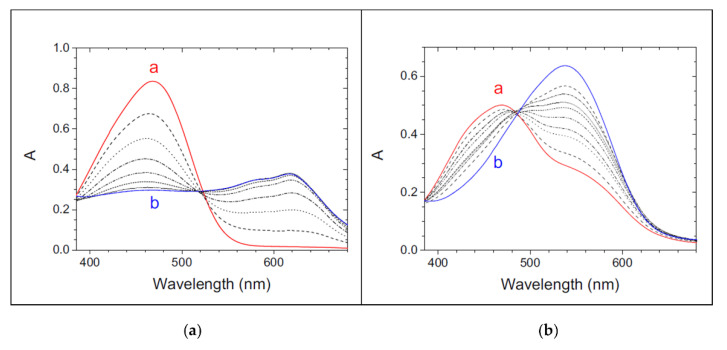
Spectral changes recorded during titration of PADA with Au^3+^; C*_Au_* from 0 M (a, red line) to 1.0 × 10^−3^ M (b, blue line), C_PADA_ = 3.0 × 10^−5^ M, C_NaCl_ = 0.03 M, T = 25 °C. (**a**) Water, pH 7.9 (0.03 M phosphate buffer), (**b**) SDS 0.02 M, pH 7.9. Figure adapted with permission from reference [80]. Copyright 2016 Elsevier.

In addition to the p*K_A_* studies, UV-Vis may also be the technique of choice in analysis of the metal-complex formation. As a first test, the simple repetition of metal-ligand titrations in water or micellar media immediately gives information on the main features of the system. Dramatically different spectral signatures unequivocally indicate reactions at the micellar phase (Figure 5). 

**Figure 6:**
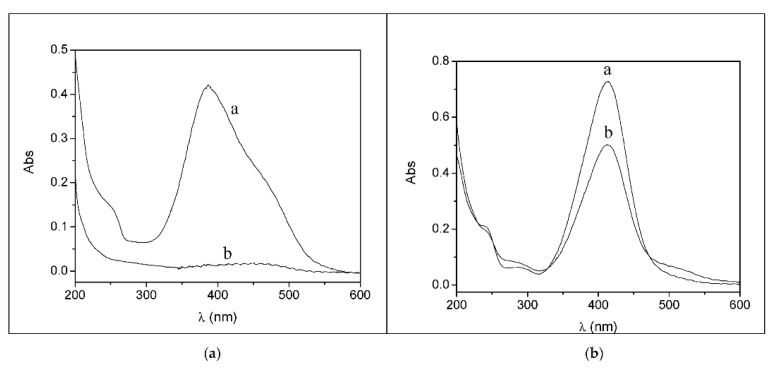
Ultrafiltration experiments on the PAR/SDS system: (**a**) absorption spectra of PAR (2.2 × 10^−5^ M) in water (line a) and of the permeate obtained after ultrafiltration in 0.040 M SDS (line b) at pH 3.2; (**b**) absorption spectra of PAR (2.2 × 10^−5^ M) in water (line a) and of the permeate obtained after ultrafiltration in 0.040 M SDS (line b) at pH 8. Figure adapted with permission from reference [77]. Copyright 2008 American Chemical Society.

Ultrafiltration experiments coupled with the spectrophotometric determination of the ligand content may be used to further confirm the extent of retention of this reactant on the surfactant (Figure 6).

Figure 6 shows that at a low pH, the ligand is 100% retained and thus that data will refer to a system reacting at this phase only, whilst this is no longer true at a higher pH. On the whole, the pH-range conditions for mechanistic studies on the solution equilibria have to be carefully tuned to possibly avoid intricate deconvolution problems from different contributions. 

**Figure 7:**
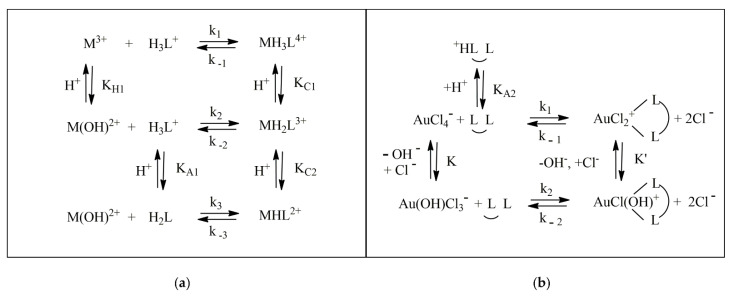
Example of (**a**) a general reaction mechanism where hydroxo species are supposed to be active together with the possible protonation of the ligand and (**b**) the case of chloride-dependent mechanisms in the presence of Au^3+^ salts. Figure 7a adapted with permission from reference [77]. Copyright 2008 American Chemical Society. Figure 7b adapted with permission from reference [80]. Copyright 2016 Elsevier.

Spectrophotometric titrations (or spectroscopic kinetic experiments) may be carried out under different conditions of the microemulsion composition, H^+^ concentration and even pressure or salt content. The latter option is needed when we seek to unveil the speciation for metal ions that may contain, for instance, chloride-coordinated forms. Fast-reaction kinetic experiments (using the stopped-flow technique) [80,81] are also precious to discern the detailed reaction mechanisms, such as those in the examples reported in Figure 7. 

The particular case of pressure dependence needs peculiar apparatuses, which enable us to make changes over a quite wide range (until 1500 atm) [82]. The value of the activation volume (ΔV^#^) can be obtained from the slope of a plot of the natural logarithm of the rate constant over pressure; these values are crucial to unveil the fine details of the metal/ligand interaction in the different media and decide between an associative or dissociative metal ion-binding mechanism (for Ni^2+^/PADA system, see, for instance, reference [83]). Note that the same study highlights that some of the most common buffers (e.g., TRIS, tris(hydroxymethyl)aminomethane) may not be inert toward the reaction (TRIS coordinates Ni^2+^).

The dependence on the experimental conditions of the overall/apparent thermodynamic parameters quantitatively enables the numerical evaluation of the binding and rate constants and qualitatively indicates the system speciation in the micellar medium. 

Studies on Ni^2+^/PADA binding in isooctane/polyoxyethylenglycol dodecyl ether/water microemulsions (polyoxyethylenglycol dodecyl ether = Brij30, non-ionic surfactant) demonstrated that the metal ion binding to the ligand was influenced by the microemulsion composition [84]. The analysis of the apparent binding constant dependence on the composition provided the distribution constants of the reactants and the real complexation constant at the interface. The results (the complex is less stable in microemulsion), once a change in the reaction mechanism is discarded, are discussed in the frame of a more efficient hydration of the nickel hexahydrate coordination complex due to the interaction between the polar head group of the surfactant and the interfacial water. Kinetic analysis of the same system further examined the changes in the rate constants as a balance between two opposite effects: the lower electrophilic character for water at the interface of the less polar Brij30 and the nucleophilicity increase due to hydrogen bonds breaking with respect to bulk [85]. 

**Figure 8:**
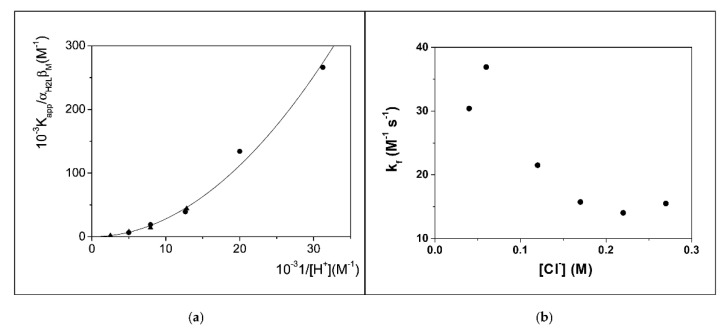
(**a**) Dependence of the apparent equilibrium constant K_app_ on the acid concentration at 25 °C for the Ga^3+^/8-hydroxyquinoline system in the presence of SDS 0.02 M; (circles) data from spectrophotometric titrations; (triangles) data from kinetic measurements (k_f_/k_d_). Figure adapted with permission from reference [86]. Copyright 2008 American Chemical Society. (**b**) Dependence of the forward-binding rate-constants on the Cl^−^ concentration for the Au^3+^-PADA system; C_DTAC_ = 0.02 M, pH 6, 25 °C. Figure adapted with permission from reference [87]. Copyright 2015 Elsevier.

In the case of the Ga^3+^/8-hydroxyquinoline system, the presence of a parabolic trend in the plot of K_app_/α_H__2__L_β_M_ over 1/[H^+^] (where α_H__2__L_ refers to the fraction of the main ligand form H_2_L^+^ over the total amount of non-complexed ligand, α_H__2__L_ = [H_2_L^+^]/L_f_, whereas β_M_ refers to the fraction of the metal ion main form M^3+^ over the total amount of uncomplexed metal, β_M_ = [M^3+^]/M_f_) indicates that the main path for complex formation involves the release of two H^+^ ions (Figure 8a). The zero intercept represents a negligible contribution on any [H^+^] independent path. On the other hand, a complex dependence of the binding parameters on the salt content (Figure 8b) unequivocally denotes the non-negligible role of some chloride coordination/expulsion in the active reaction paths.

These studies shed light on the differences in the speciation profiles for both the ligand and the metal ion when switching from water to the micellar phase. This strongly affects the behavior and the main properties of the system and is crucial to be known for any practical application. 

The micellar medium can also strongly alter the main reacting species in the case of the metal ion. For the Pd^2+^/PADA system, in the studied pH range, PADAH^+^, PADAH_2_^2+^, PdCl_4_^2−^ and Pd(H_2_O)Cl_3_^−^ are the main species in water, whereas neutral PADA and a new reaction path involving the hydroxo species PdCl_3_OH^2−^ need to be involved in the reaction mechanism in DTAC micellar solutions [76]. In the case of gold, a study of the Au^3+^/PADA system showed that the aquo-species Au(H_2_O)Cl_3_ and Au(OH)_3_(H_2_O), which are not involved in the binding process in pure water, play an important role in the H_2_O/SDS medium [77]. A study of the reaction mechanism of the same Au^3+^/PADA system in the H_2_O/DTAC medium demonstrated that Au(H_2_O)Cl_3_ and Au(H_2_O)_2_Cl_2_^+^ play the major roles here [87]. In the case of the Ga^3+^/8-hydroxyquinoline system, the Ga^3+^ ion is stabilized and the GaHL bound form appears in SDS media (GaL in water) [86]. 

Determination of the speciation on the water/micellar pseudo phase also enables us to finetune the reactivity of the system in the presence of metal ion mixtures. For instance, Au^3+^ may be efficiently extracted, recovered and separated from Cu^2+^ by using a step-by-step procedure based on either DTAC or SDS thanks to the tendency of gold to form negatively charged species [88]. This also holds for the Pd^2+^/Pt^2+^mixture [89]. In addition, a similar MEUF (micellar-enhanced ultrafiltration) approach may be used to simultaneously remove metal ions and organic contaminants from wastewater. For instance, Cd^2+^ (ca. 90%) and phenol (ca. 40%) may be efficiently extracted by a carefully calibrated mixture of Triton X-100 and SDS [90]. A similar study on the Cd^2+^/phenol system in Brij35/SDS mixtures evidenced the possibility of optimizing the extraction procedure until achieving more than 90% recovery of both species [91].

These studies may be much trickier than they appear as it is not straightforward that the reactions will occur either in the micelle *or* in the bulk; they could occur in both phases [92]. Suitable thermodynamic and/or kinetic models will be needed to correctly analyze these complicated equilibria [93]. The pseudo-phase ion-exchange (PIE) model is a widely applied extension of the pseudo-phase model (PP) and supposes that: (i) water and micelle are two separate phases in which the reactants are distributed with very fast kinetics; (ii) the reaction in the micellar pseudo phase occurs mainly at the micelle surface; (iii) charged inert ions may compete at the charged micellar surface but the association degree of counter ions remains constant. Sometimes, it has been recommended that, when counter ions are very hydrophilic, PIE is not totally correct and mass-action models are necessary [73].

Under conditions where the reactants do distribute over the two phases, the binding constants measured experimentally by spectrophotometric data become apparent values. Any apparent binding (or kinetic) constant obtained also becomes a function of the retention% of the reactants. The apparent equilibrium constant, K*_app_*, will be related to the binding reaction, as usually written for a one-phase-only system (Equation (12))

M_f_ + L_f_ ⇆ ML_T_ (12)

where M_f_ and L_f_ are the total concentrations of uncomplexed metal and ligand, while ML_T_ is the total concentration of complex which, in this simplified example, is supposed to be at a 1:1 stoichiometric ratio. Thus, in the presence of surfactant, the expression for *K_app_* becomes

(13) $$K_{app}=\frac{\left([ML_{T}^{S\;}\right]\;+\;[ML_{T}^{W}])}{\left([M_{f}^{S\;}\right]\;+\;\left[M_{f}^{W}\right])\left({[L}_{f}^{S\;}\right]\;+\;\left[L_{f}^{W}\right])}$$

where the subscripts *W* and *S* denote the concentrations of the reactants in the aqueous and surfactant pseudo-phases, respectively. The extent of adsorption on the micelles (R) may be evaluated by ultrafiltration experiments where, after separation of retentate and permeate, a spectrum of the permeate is compared with that of the initial solution (see Figure 5). Then, Equation (14) is used to calculate the retention coefficient *R_i_* for each *i*th species of the system

(14) $$R_{i}=\frac{C_{i}^{W}}{C_{i}^{S}}$$

where *C_i_^W^* and *C_i_^S^* are the molar concentrations in water and in the surfactant, respectively. On the basis of these latter two equations, it can be demonstrated that Equation (15) holds; a similar procedure can be applied to the kinetic constants [94].

(15) $$K_{app}^{S}=\frac{\;\;\;\;[ML_{T}^{S}]}{\;\left[M_{f}^{S}]\;[L_{f}^{S}\right]}=\left(K_{app}-\frac{K_{app}^{W}}{\left(1+\frac{1}{R_{Mf}}\right)\left(1+\frac{1}{R_{Lf}}\right)}\right)\left(1+R_{Mf}\right)\left(1+R_{Lf}\right)$$

Such a procedure was applied, for instance, in the case of the study of Ni^2+^/hydroxamic acid systems in the presence of SDS. Analysis of the binding mechanism, obtained by carefully splitting the contributions, provided a picture where the Ni^2+^ + HL ⇄ NiHL^2+^ step occurs in both pseudo-phases, whereas the Ni^2+^ + L^−^ ⇄ NiL^+^ step in water is replaced by NiOH^+^ + HL ⇄ NiL^+^ when in SDS [94]. A similar procedure was applied in the case of the Cu^2+^/tartaric acid systems in the presence of non-ionic Brij 58 micelles [95]; this detailed thermodynamic study indicated that the structures of the complexes incorporated in the micelles and the species-distribution diagrams are modified if compared to water and allowed to model the evolution of the extraction yields of Cu^2+^ ions by the micelles as a function of pH. 

What has been already discussed for [H^+^] concentration changes in the micellar pseudo-phase also holds for the reactants. Consequently, a further main point is that the presence of a micellar/vesicular system offers a limited place where species may be condensed, obtaining local concentrations that are much higher than those in the bulk solvent. This produces the so-called “catalytic effect”, which refers to catalysis only in the sense that the rate constant for the association path is enhanced by the concentration increase on the micelle surface [75]. The effect on the dissociation rate constant is often much smaller, and therefore, the apparent association constant will increase. This effect is maximized just above the critical aggregation concentration (or critical micellar concentration, cmc). If the surfactant concentration rises, the number of micellar reactors progressively increases. Thus, the reactants are diluted and the rate and equilibrium constant enhancements are lost and leveled off. To obtain the best enhancement effect, negatively charged reactors (SDS) are used for positively charged metal ions (for instance, Cd^2+^ or Ni^2+^) [75]; however, since the metal center may be present as a negative species [76,87], conversely, a positive micelle (e.g., DTAC) becomes the best choice. The “catalytic effect” was also found to occur for the Ni^2+^/PADA system in the presence of zwitterionic micelles formed with carboxybetaines; nevertheless, it does not occur for zwitterionic micelles by sulfobetaines because metal ions are not appreciably adsorbed by them [96]. The effect may also be lost if the species are compartmentalized in different parts of the amphiphilic system: again, if the ligand is highly hydrophobic it may be preferentially located in the core of the micelle, far from the surface. For instance, the partition coefficient for 1-pyridyl-2-azo-2-naphthol (PAN) between the micelle surface and the core was found to be *K*_PAN_ = [PAN]_s_/[PAN]_c_ = 0.05 where “s” represents the micelle surface site (reactive) and “c” is the micelle core (unreactive) [97]. Finally, even a so-called “anti-catalytic effect” may be observed when the micellar surface bears the same charge as the metal species (for instance, gold aquo/chloro complexes in SDS) [77]. 

**Figure 9:**
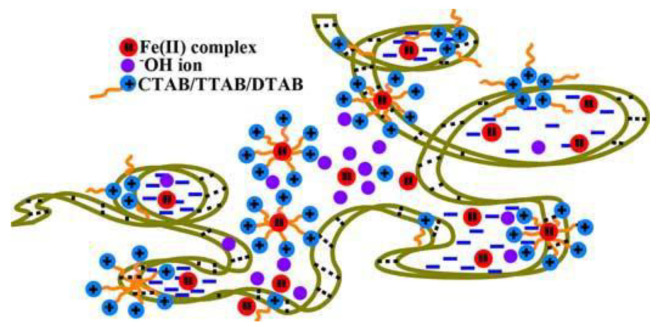
Cartoon showing the positioning of reactant species on guar-gum segments in gum aqueous solution. Figure adapted with permission from reference [98]. Copyright 2018 Taylor & Francis.

Catalytic or anti-catalytic effects can also occur in the presence of more complicated micro-heterogeneous ternary environments. For instance, the kinetics of alkaline hydrolysis of Fe^2+^/phenanthroline metal complexes changes were studied in the presence of surfactants/guar-gum mixtures [98]. The observed pseudo-first-order rate-constant (k_obs_) values were obtained from the decrease in the absorbance at 510 nm versus the time under pseudo-first-order conditions ([OH^−^] >> [complex]). Longer hydrophobic chains better catalyze the hydrolysis reaction (the surfactant was CH_3_-[CH2]_n_-CH_2_-N(CH_3_^)^_3_^+^, cetyltrimethylammonium/CTAB/*n* = 14 > tetradecyltrimethylammonium/TTAB/*n* = 12 > dodecyltrimethylammonium/DTAB/*n* = 10). However, the binding to guar gum by the surfactant also follows the order CTAB > TTAB > DTAB. On the whole, it was found that the rate constant is k_1_(DTAB-guar gum) >> k_1_(TTAB-guar gum) >> k_1_(CTAB-guar gum) >> k_1_(aqueous medium) >> k_1_(in aqueous gum medium). These data were discussed on the basis of gum-amphiphile interactions as a function of the hydrophobic chain length of amphiphiles (Figure 9).

## 4. Computational Approaches to Examine Metal-Based Compound/Amphiphile Interactions 

The interaction of metal ions and metal compounds with amphiphilic systems has also been tackled by computational chemistry approaches, with the aim to shed light on the atomic-level binding sites and mechanisms involved and to provide support to improve properties such as solubility and stability in aqueous solutions. However, the literature search performed highlights that, despite the usefulness of such approaches, they have not yet been largely used by specialists in this research field and very few references can be found.

Typically, for such computational investigations, molecular dynamics (MD) simulations, using empirical force fields, are used to model the formation of large amphiphilic aggregates, involving thousands of atoms and the presence of an explicit water solvent, and to check how the presence of small molecules, metal ions or metal compounds, in our case, perturbs the overall system, also highlighting the preferential interactions between aggregates and metal compounds. Quite often, MD simulations have been used as a support for the structural and dynamic interpretation of experimental data, such as from NMR spectroscopy or neutron-scattering structural investigations.

Furthermore, quantum chemistry approaches, essentially using density functional theory (DFT) calculations, have been used to investigate the structure of isolated amphiphilic molecules or their small aggregates, to obtain information on their polarity properties and on their binding sites and binding strength with metal compounds.

### 4.1. DFT Calculations

**Figure 10:**
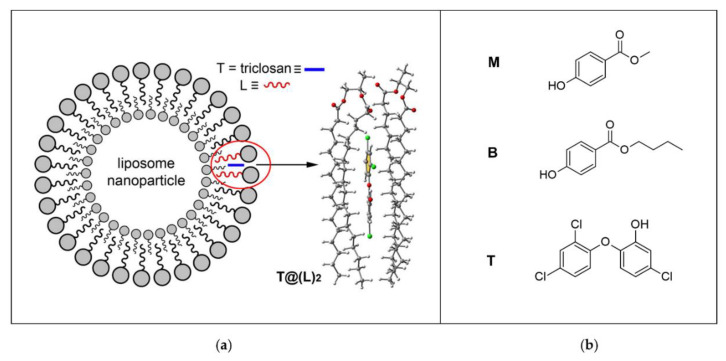
(**a**) Schematic representation of the liposomal nanoparticle and in-silico DFT model of the complex of a dimer of the hydrophobic acyl chain region of the phospholipid with triclosan (T@(L)_2_); (**b**) target contaminants explored: methylparaben (M), butylparaben (B) and triclosan (T). Figure adapted with permission from reference [102]. Copyright 2016 American Chemical Society.

Singh recently reported a short review focused on the applications of DFT for drug design based on the investigation of drug-biomembrane interaction, aimed at proposing structure changes to improve biomembrane crossing and to enhance the drug efficacy profile [99]. Arslan et al. [100] obtained, by DFT calculations, the octanol-water partition coefficients of drug-like molecules, with the aim to compare the Gibbs solvation free energies of potential drug candidates with experimentally available LogP values. In detail, the n-octanol/water partition coefficient, LogP or log K_ow_, describes the drug hydrophobicity and membrane permeability, a theoretically determined basic property for drug design, using the combinations of three functionals, two basis sets, an implicit salvation method with one explicit water molecule and performing a conformational search by using the semi-empirical PM3 method. The results obtained provide suggestions on the methodology that better reproduces the experimental data. DFT calculations have also been exploited by Popovic-Nikolic et al. [101], to theoretically investigate the ionization of three angiotensin II receptor blockers, also known as sartans, two ampholytes and one diacid molecules, all in aqueous solution and in the presence of surfactant micelle models. p*K_a_* values have been calculated using implicit salvation methods and compared with the corresponding experimental data. Moreover, structural information on the binding to the micelle surfaces was provided for the equilibrium forms of the selected sartan molecules. In this study, however, the micelles were not explicitly considered. The interaction of sartans with micelles was estimated by the Connolly accessible area descriptor, defined as the surface area made by the solvent sphere, which influences the hydrophobic interactions of a molecule. Finally, Oliver et al. [102] used dispersion-corrected DFT calculations to study the noncovalent binding of three emerging contaminants with two dipalmitoylphosphatidylcholine molecules, used as a model of a phospholipid membrane, to determine the binding energies and the structures of the contaminant-phospholipid complexes, with the aim to estimate the degree of penetration of the guest across the lipid bilayer and to predict the potential bioavailability and toxicity (Figure 10). Solvent effects were mimicked by using both discrete water molecules in the polar head of the surfactant molecule and an implicit solvation model.

### 4.2. MD Simulations

John et al. [103] investigated cation-induced lipid membrane movement. In detail, a lipid membrane model system, composed of a differently charged free-floating bilayer, in the presence and absence of Ca^2+^ and Na^+^, adjacent to a self-assembled monolayer (SAM), was investigated by neutron reflectivity and quartz crystal microbalance measurements. The experimental results were interpreted with the support of MD simulations to determine the cation and water interfacial distribution in the membrane model.

With the aim to explore the ability of lipids and proteins to shape realistic membrane models, Pezeshkian and Marrink [104] used both coarse-grained MD and all-atom MD simulations, which were combined to examine the dynamic properties of realistic membrane shapes, thus obtaining information on protein-lipid interactions related to cellular morphologies of entire organelles. Kinnun et al. [105] modelled small-angle neutron scattering (SANS) data by MD simulations to study the supramolecular structures of cell membranes. Interestingly, their investigation allowed the researchers to determine the location of cholesterol in bilayer membrane models. Tsukanov et al. [106] studied the interaction of bimetallic Ag–Cu nanoparticles (Cu_70_Ag_30_, Ag_70_Cu_30_ and Cu_70_Ag_30_O_4_) with phospholipid and lipopolysaccharide membranes by MD simulations. In this study, DFT calculations were also used to estimate the composition-dependent surface-charge density of the Ag-Cu nanoparticle. Finally, Pujol-Giménez et al. [107] used a combination of molecular dynamic simulations and p*Ka* calculations to discover relevant residues in the human divalent metal transporter 1 (hDMT1) protein, a divalent metal ion transporter that contributes to H^+^/Fe^2+^ coupled transport.

## 5. Conclusions

In this review, we summarized the literature contributions to the quantitative investigation of the binding processes involving metal-based compounds and surfactants. Different techniques and methods have been discussed including isothermal titration calorimetry, spectrophotometry, DFT calculations and MD simulations.

We found only a few papers reporting the binding process of metal complexes and amphiphilic-based aggregates through isothermal titration calorimetry experiments, despite the fact crucial information on the thermodynamic profiles of the species in solution may be easily obtained by this technique. Calorimetry was also used to explore the direct interaction of metal ions with models of biomembranes made of amphiphilic molecules, thus obtaining quantitative data on the driving forces involved in the binding events. We also analyzed the spectrophotometric investigations on the metal-ligand equilibria in amphiphilic media to determine both thermodynamic and kinetic parameters in solution. Since the application of surfactants in metal complexation and extraction is a widely reported topic, the accurate differences observed in the speciation profiles of both the ligands and the metal ions from simple water to the micellar phase should be taken into account. Finally, we reviewed some works on computational chemistry approaches to study the interaction of metal ions with amphiphilic systems that investigate, at the atomic level, the binding sites and the mechanisms involved in the recognition processes.

More detailed studies will be beneficial to completely characterize the complex multicomponent systems examined in this work since only a thorough quantitative analysis of data from different techniques/approaches may provide key insights into the binding processes, affinities, species, driving forces and interactions concerning metal-based compounds and amphiphilic molecules. The ultimate goal is to better understand the mechanisms of phenomena involving biologically relevant metal ions and to promote the design and development of metal/surfactants-based systems that may efficiently operate in drug delivery as well as in water remediation.

## Abbreviations

The following abbreviations are used in this manuscript:

BDHAC	Benzyldimethylhexadecylammonium
Brij 30	Polyoxyethylenglycol dodecyl ether
Brij 58	Polyoxyethylenglycol hexadecyl ether
CPC	Hexadecylpyridinium chloride
CTAB	Hexadecyltrimethylammonium bromide
CTAC	Hexadecyltrimethylammonium chloride
DFT	Density functional theory
DMPA	1, 2-dimyristoyl-sn-glycero-3-phosphatidic acid
DOPC	Dioleoylphosphatidylcholine
DOPG	Dioleoylphosphatidylglycerol
DPPC	Dipalmitoyl-phosphatidylcholine
DTAB	Dodecyltrimethylammonium bromide
DTAC	Dodecyltrimethylammonium chloride
FRET	Fluorescence resonance energy transfer
Gd-DTPA	Gadolinium-diethylenetriamine penta-acetic acid
HCPT	10-hydroxycamptothecine
hCtr	Human copper transporters
hDMT	Human divalent metal transporter
HP	HCPT-peptide
ITC	Isothermal titration calorimetry
LUV	Large unilamellar vesicle
LMWF_8775_	Low molecular weight fucoidan
M2-motif	Second methionine-rich motif
MD	Molecular dynamics
MEUF	Micellar-enhanced ultrafiltration
MR	Magnetic resonance
MRI	Magnetic resonance imaging
PADA	Pyridine-2-azo-p-dimethylaniline
PAN	1-pyridyl-2-azo-2-naphthol
PAR	4-(2-pyridylazo)resorcinol
PCplasm	Phosphatidylcholine plasmalogen
PEplasmas	Phosphatidylethanolamine plasmalogen
PIE	Polyoxyethylenglycol dodecyl ether
POPC	Palmitoyloleoylphosphatidylcholine
PP	Pseudo-phase model
SANS	Small-angle neutron scattering
SDP	Sodium dodecylphosphonate
SDS	Sodium dodecyl sulfate
SL	Sodium laurate
SO	Sodium oleate
TMD	Transmembrane domain
TPP_1880_	Thermolysin-hydrolyzed protamine peptide
TRIS	Tris(hydroxymethyl)aminomethane
TTAB	Tetradecyltrimethylammonium

## References

[1] Zoroddu M.A., Aaseth J., Crisponi G., Medici S., Peana M., Nurchi V.M. (2019). The essential metals for humans: A brief overview. J. Inorg. Biochem..

[2] Melnikov P., Zanoni L.Z. (2010). Clinical effects of cesium intake. Biol. Trace Elem. Res..

[3] Vashishtha A.K., Wang J., Konigsberg W.H. (2016). Different divalent cations alter the kinetics and fidelity of DNA polymerases. J. Biol. Chem..

[4] Yang Z., Hayes J.J. (2011). The divalent cations Ca^2+^ and Mg^2+^ play specific roles in stabilizing histone–DNA interactions within nucleosomes that are partially redundant with the core histone tail domains. Biochemistry.

[5] Smith A.M., Hardy J.G., Schacher F.H. (2015). CHAPTER 1: Interaction of Metal Ions with Proteins as a Source of Inspiration for Biomimetic Materials. Functional Metallosupramolecular Materials.

[6] https://www.epa.gov/caddis-vol2/metals.

[7] Li Z., Ma Z., van der Kuijp T.J., Yuan Z., Huang L. (2014). A review of soil heavy metal pollution from mines in China: Pollution and health risk assessment. Sci. Total Environ..

[8] Järup L. (2003). Hazards of heavy metal contamination. Br. Med. Bull..

[9] Friberg L., Mottet N.K. (1989). Accumulation of methylmercury and inorganic mercury in the brain. Biol. Trace Elem. Res..

[10] Rice K.M., Walker E.M., Wu M., Gillette C., Blough E.R. (2014). Environmental mercury and its toxic effects. J. Prev. Med. Public Health.

[11] Guzzi G., Ronchi A., Pigatto P. (2021). Toxic effects of mercury in humans and mammals. Chemosphere.

[12] Jan A.T., Azam M., Siddiqui K., Ali A., Choi I., Haq Q.M. (2015). Heavy metals and human health: Mechanistic insight into toxicity and counter defense system of antioxidants. Int. J. Mol. Sci..

[13] Huat T.J., Camats-Perna J., Newcombe E.A., Valmas N., Kitazawa M., Medeiros R. (2019). Metal toxicity links to Alzheimer’s disease and neuroinflammation. J. Mol. Biol..

[14] Lazarević T., Rilak A., Bugarčić Ž.D. (2017). Platinum, palladium, gold and ruthenium complexes as anticancer agents: Current clinical uses, cytotoxicity studies and future perspectives. Eur. J. Med. Chem..

[15] Florea A.M., Büsselberg D. (2011). Cisplatin as an anti-tumor drug: Cellular mechanisms of activity, drug resistance and induced side effects. Cancers.

[16] Thota S., Rodrigues D.A., Crans D.C., Barreiro E.J. (2018). Ru (II) compounds: Next-generation anticancer metallotherapeutics?. J. Med. Chem..

[17] Sun Q., Li Y., Shi H., Wang Y., Zhang J., Zhang Q. (2021). Ruthenium Complexes as Promising Candidates against Lung Cancer. Molecules.

[18] Guarra F., Pratesi A., Gabbiani C., Biver T. (2021). A focus on the biological targets for coinage metal-NHCs as potential anticancer complexes. J. Inorg. Biochem..

[19] Ndagi U., Mhlongo N., Soliman M.E. (2017). Metal complexes in cancer therapy–an update from drug design perspective. Drug Des. Dev. Ther..

[20] Sambhy V., MacBride M.M., Peterson B.R., Sen A. (2006). Silver bromide nanoparticle/polymer composites: Dual action tunable antimicrobial materials. J. Am. Chem. Soc..

[21] Aderibigbe B.A. (2017). Metal-based nanoparticles for the treatment of infectious diseases. Molecules.

[22] Ahmad M.Z., Akhter S., Jain G.K., Rahman M., Pathan S.A., Ahmad F.J., Khar R.K. (2010). Metallic nanoparticles: Technology overview & drug delivery applications in oncology. Expert Opin. Drug Deliv..

[23] Savić S., Tamburić S., Savić M.M. (2010). From conventional towards new–natural surfactants in drug delivery systems design: Current status and perspectives. Expert Opin. Drug Deliv..

[24] Shah A., Shahzad S., Munir A., Nadagouda M.N., Khan G.S., Shams D.F., Dionysiou D.D., Rana U.A. (2016). Micelles as soil and water decontamination agents. Chem. Rev..

[25] Rasheed T., Shafi S., Bilal M., Hussain T., Sher F., Rizwan K. (2020). Surfactants-based remediation as an effective approach for removal of environmental pollutants—A review. J. Mol. Liq..

[26] Arena G., Sgarlata C., Atwood J.L. (2017). Modern Calorimetry: An Invaluable Tool in Supramolecular Chemistry. Comprehensive Supramolecular Chemistry II.

[27] Ghai R., Falconer R.J., Collins B.M. (2012). Applications of isothermal titration calorimetry in pure and applied research—Survey of the literature from 2010. J. Mol. Recognit..

[28] Vega S., Abian O., Velazquez-Campoy A. (2015). A unified framework based on the binding polynomial for characterizing biological systems by isothermal titration calorimetry. Methods.

[29] Du X., Li Y., Xia Y.L., Ai S.M., Liang J., Sang P., Ji X.-L., Liu S.Q. (2016). Insights into protein–ligand interactions: Mechanisms, models, and methods. Int. J. Mol. Sci..

[30] Sgarlata C., Schneider B.L., Zito V., Migliore R., Tegoni M., Pecoraro V.L., Arena G. (2021). Lanthanide Identity Governs Guest-Induced Dimerization in Ln^III^[15-MC_CuIIN(L-pheHA)_-5])^3+^ Metallacrowns. Chem. Eur. J..

[31] Bonaccorso C., Migliore R., Volkova M.A., Arena G., Sgarlata C. (2017). Self-assembling of supramolecular adducts by sulfonato-calix[4]arene and pyridinium gemini guests in neutral aqueous solution. Thermochim. Acta.

[32] Bonaccorso C., Brancatelli G., Forte G., Arena G., Geremia S., Sciotto D., Sgarlata C. (2014). Factors driving the self-assembly of water-soluble calix [4] arene and gemini guests: A combined solution, computational and solid-state study. RSC Adv..

[33] Claveria-Gimeno R., Vega S., Abian O., Velazquez-Campoy A. (2017). A look at ligand binding thermodynamics in drug discovery. Expert Opin. Drug Discov..

[34] Loh W., Brinatti C., Tam K.C. (2016). Use of isothermal titration calorimetry to study surfactant aggregation in colloidal systems. Biochim. Biophys. Acta Gen. Subj..

[35] Archer W.R., Schulz M.D. (2020). Isothermal titration calorimetry: Practical approaches and current applications in soft matter. Soft Matter.

[36] Choudhary S., Talele P., Kishore N. (2015). Thermodynamic insights into drug–surfactant interactions: Study of the interactions of naporxen, diclofenac sodium, neomycin, and lincomycin with hexadecytrimethylammonium bromide by using isothermal titration calorimetry. Colloids Surf. B Biointerfaces.

[37] Mukhija A., Kishore N. (2017). Partitioning of drugs in micelles and effect on micellization: Physicochemical insights with tryptophan and diclofenac sodium. Colloids Surf. A Physicochem. Eng. Asp..

[38] Banipal T.S., Kaur H., Banipal P.K. (2017). Studies on the binding ability of diclofenac sodium to cationic surfactants micelles in aqueous ethanol solutions. J. Therm. Anal. Calorim..

[39] Kaur R., Rani A., Banipal P.K., Banipal T.S. (2019). Study on interactions of vitamin B1 with sodium dodecyl sulfate for potential food applications: Conductometric, volumetric, calorimetric and spectroscopic approach. J. Mol. Liq..

[40] Dasgupta M., Judy E., Kishore N. (2020). Partitioning of anticancer drug 5-fluorouracil in micellar media explored by physicochemical properties and energetics of interactions: Quantitative insights for implications in drug delivery. Colloids Surf. B Biointerfaces.

[41] Le V.H., Buscaglia R., Chaires J.B., Lewis E.A. (2013). Modeling complex equilibria in isothermal titration calorimetry experiments: Thermodynamic parameters estimation for a three-binding-site model. Anal. Biochem..

[42] Ikonen M., Murtomäki L., Kontturi K. (2010). Microcalorimetric and zeta potential study on binding of drugs on liposomes. Colloids Surf. B Biointerfaces.

[43] Migliore R., Granata G., Rivoli A., Consoli G.M.L., Sgarlata C. (2021). Binding affinity and driving forces for the interaction of calixarene-based micellar aggregates with model antibiotics in neutral aqueous solution. Front. Chem..

[44] Migliore R., D’Antona N., Sgarlata C., Consoli G.M.L. (2021). Co-Loading of Temozolomide and Curcumin into a Calix [4] arene-Based Nanocontainer for Potential Combined Chemotherapy: Binding Features, Enhanced Drug Solubility and Stability in Aqueous Medium. Nanomaterials.

[45] Arena G., Gans P., Sgarlata C. (2016). HypCal, a general-purpose computer program for the determination of standard reaction enthalpy and binding constant values by means of calorimetry. Anal. Bioanal. Chem..

[46] Cheng T.M., Li R., Kao Y.C.J., Hsu C.H., Chu H.L., Lu K.Y., Changou C.A., Chang C.C., Chang L.H., Tsai M.L. (2020). Synthesis and characterization of Gd-DTPA/fucoidan/peptide complex nanoparticle and in vitro magnetic resonance imaging of inflamed endothelial cells. Mater. Sci. Eng. C.

[47] Cai Y., Shen H., Zhan J., Lin M., Dai L., Ren C., Shi Y., Liu J., Gao J., Yang Z. (2017). Supramolecular “Trojan Horse” for nuclear delivery of dual anticancer drugs. J. Am. Chem. Soc..

[48] Xiao H., Song H., Zhang Y., Qi R., Wang R., Xie Z., Huang Y., Li Y., Wu Y., Jing X. (2012). The use of polymeric platinum (IV) prodrugs to deliver multinuclear platinum (II) drugs with reduced systemic toxicity and enhanced antitumor efficacy. Biomaterials.

[49] Eisenberg M., Gresalfi T., Riccio T., McLaughlin S. (1979). Adsorption of monovalent cations to bilayer membranes containing negative phospholipids. Biochemistry.

[50] Ohki S., Ohshima H. (1999). Interaction and aggregation of lipid vesicles (DLVO theory versus modified DLVO theory). Colloids Surf. B Biointerfaces.

[51] Claessens M.M.A.E., Leermakers F.A.M., Hoekstra F.A., Cohen Stuart M.A. (2007). Opposing effects of cation binding and hydration on the bending rigidity of anionic lipid bilayers. J. Phys. Chem. B.

[52] Klasczyk B., Knecht V., Lipowsky R., Dimova R. (2010). Interactions of alkali metal chlorides with phosphatidylcholine vesicles. Langmuir.

[53] Maity P., Saha B., Kumar G.S., Karmakar S. (2016). Binding of monovalent alkali metal ions with negatively charged phospholipid membranes. Biochim. Biophys. Acta-Biomembr..

[54] Kerek E., Hassanin M., Zhang W., Prenner E.J. (2017). Preferential binding of Inorganic Mercury to specific lipid classes and its competition with Cadmium. Biochim. Biophys. Acta-Biomembr..

[55] Chang L.W. (1977). Neurotoxic effects of mercury—A review. Environ. Res..

[56] Méndez-Armenta M., Ríos C. (2007). Cadmium neurotoxicity. Environ. Toxicol. Pharmacol..

[57] Garidel P., Blume A. (2019). Electrostatic interactions of alkaline earth cations with 1, 2-dimyristoyl-sn-glycero-3-phosphatidic acid (DMPA) model membranes at neutral and acidic pH. Eur. Biophys. J..

[58] Wang Y., Wang L., Li F. (2013). Micelle-bound structure of an extracellular Met-rich domain of hCtr1 and its binding with silver. RSC Adv..

[59] Dong Z., Wang Y., Wang C., Xu H., Guan L., Li Z., Li F. (2015). Self-Assembly of the Second Transmembrane Domain of hCtr1 in Micelles and Interaction with Silver Ion. J. Phys. Chem. B.

[60] Dong Z., Guan L., Wang C., Xu H., Li Z., Li F. (2016). Reconstruction of a helical trimer by the second transmembrane domain of human copper transporter 2 in micelles and the binding of the trimer to silver. RSC Adv..

[61] Yang Y., Zhu Y., Hu H., Cheng L., Liu M., Ma G., Yuan S., Cui P., Liu Y. (2019). Cuprous binding promotes interaction of copper transport protein hCTR1 with cell membranes. Chem. Commun..

[62] Chakraborty M., Hsiao F.W., Naskar B., Chang C.H., Panda A.K. (2012). Surfactant-assisted synthesis and characterization of stable silver bromide nanoparticles in aqueous media. Langmuir.

[63] Majumder S., Naskar B., Ghosh S., Lee C.H., Chang C.H., Moulik S.P., Panda A.K. (2014). Synthesis and characterization of surfactant stabilized nanocolloidal dispersion of silver chloride in aqueous medium. Colloids Surf. A Physicochem. Eng. Asp..

[64] Wang Z., Xu S., Acosta E. (2015). Heat of adsorption of surfactants and its role on nanoparticle stabilization. J. Chem. Thermodyn..

[65] Balleza D., Mescola A., Marín–Medina N., Ragazzini G., Pieruccini M., Facci P., Alessandrini A. (2019). Complex phase behavior of GUVs containing different sphingomyelins. Biophys. J..

[66] Mescola A., Ragazzini G., Alessandrini A. (2020). Daptomycin Strongly Affects the Phase Behavior of Model Lipid Bilayers. J. Phys. Chem. B.

[67] Kaur R., Mehta S.K. (2014). Self aggregating metal surfactant complexes: Precursors for nanostructures. Coord. Chem. Rev..

[68] Wagay T.A., Dey J., Kumar S., Aswal V.K., Ismail K. (2016). Aggregation, adsorption, counterion binding, thermal and scattering behavior of metallosurfactant *cis*-[Co(en)_2_(C_12_H_25_NH_2_)Cl](NO_3_)_2_. Colloids Surf. A Physicochem. Eng. Asp..

[69] Grein F., Müller A., Scherer K.M., Liu X., Ludwig K.C., Klöckner A., Strach M., Sahl H.-G., Kubitscheck U., Schneider T. (2020). Ca^2+^-Daptomycin targets cell wall biosynthesis by forming a tripartite complex with undecaprenyl-coupled intermediates and membrane lipids. Nat. Commun..

[70] Kreutzberger M.A., Pokorny A., Almeida P.F. (2017). Daptomycin–phosphatidylglycerol domains in lipid membranes. Langmuir.

[71] Pokorny A., Khatib T.O., Stevenson H. (2018). A quantitative model of daptomycin binding to lipid bilayers. J. Phys. Chem. B.

[72] Khalid K., Zain S.M., Khan M.N. (2017). Catalytic effects of cationic nanoparticle (CTABr/NaX/H_2_O, X = Cl, Br) for the piperidinolysis of phenyl salicylate ions. Kinet. Catal..

[73] Brandariz I., Iglesias E. (2014). Micellar effects on aromatic esters hydrolysis. Colloids Surf. A Physicochem. Eng. Asp..

[74] Basu A., Saha B. (2010). Kinetic studies on hexavalent chromium reduction. Am. J. Analyt. Chem..

[75] Monteleone G., Morroni L., Robinson B., Tinè M.R., Venturini M., Secco F. (2004). Metal ion extraction in surfactant solution: Ni^2+^(aq) and Cd^2+^ (aq) with the ligands PADA and PAR in SDS micellar systems. Colloids Surf. A Physicochem. Eng. Asp..

[76] Ghezzi L., Robinson B.H., Secco F., Tiné M.R., Venturini M. (2007). Binding of Pd (II) to Pada in water/micellar system: Complex formation, kinetics in water and DTAC solution. Colloids Surf. A Physicochem. Eng. Asp..

[77] Biver T., Boggioni A., Secco F., Venturini M. (2008). Gallium (III)/4-(2-pyridylazo) resorcinol system in water and SDS solution: Kinetics and thermodynamics. Langmuir.

[78] Biver T., Aydinoglu S., Greco D., Macii F. (2018). Mechanistic details on Pd (II)/5, 10, 15, 20-tetrakis (1-methyl-4-pyridyl) porphyrin complex formation and reactivity in the presence of DNA. Monatsh. Chem..

[79] Aydinoglu S., Biver T., Secco F., Venturini M. (2014). Effects of micelle nature and concentration on the acid dissociation constants of the metal extractor PADA. Colloids Surf. A Physicochem. Eng. Asp..

[80] Aydinoglu S., Biver T., Secco F., Venturini M. (2016). The mechanism of the reaction between Au (III) and PADA in sodium dodecylsulphate. Colloids Surf. A Physicochem. Eng. Asp..

[81] Kreutzberger A.J., Pokorny A. (2012). On the origin of multiphasic kinetics in peptide binding to phospholipid vesicles. J. Phys. Chem. B.

[82] Hubbard C.D., van Eldik R. (2010). Mechanistic information on some inorganic and bioinorganic reactions from volume profile analysis. Inorg. Chim. Acta.

[83] Gazzaz H.A., Ember E., Zahl A., van Eldik R. (2009). Mechanistic information from volume profiles for water exchange and complex-formation reactions of aquated Ni (II). pH, buffer and medium effects. Dalton Trans..

[84] Cabaleiro-Lago C., Garcia-Rio L., Hervés P., Pérez-Juste J. (2007). Spectrophotometric study of metal–ligand reactions in isooctane/Brij30/water nonionic microemulsions. Colloids Surf. A Physicochem. Eng. Asp..

[85] Cabaleiro-Lago C., Garcia-Rio L., Hervés P., Pérez-Juste J. (2007). Nonionic microemulsions: Effects of the interface on metal–ligand reactions *Colloids Surf*. A Physicochem. Eng. Asp..

[86] Biver T., Ghezzi L., Malvaldi V., Secco F., Tiné M.R., Venturini M. (2009). Kinetics and equilibria of the interaction of 8-hydroxyquinoline with gallium (III) in water and sodium dodecyl sulfate solution. J. Phys. Chem. B.

[87] Aydinoglu S., Biver T., Secco F., Venturini M. (2015). The kinetics of gold (III) extraction by pyridine-2-azo-p-dimethylaniline in water and in micellar systems. Colloids Surf. A Physicochem. Eng. Asp..

[88] Aydinoglu S., Biver T., Ceccarini A., Secco F., Venturini M. (2015). Gold (III) extraction and recovery and gold (III)/copper (II) separation using micelles. Colloids Surf. A Physicochem. Eng. Asp..

[89] Biver T., Paoletti C., Secco F., Venturini M. (2014). Extraction, separation and recovery of palladium and platinum by a kinetic method combined with ultrafiltration. Colloids Surf. A Physicochem. Eng. Asp..

[90] Li X., Zeng G.M., Huang J.H., Zhang D.M., Shi L.J., He S.B., Ruan M. (2011). Simultaneous removal of cadmium ions and phenol with MEUF using SDS and mixed surfactants. Desalination.

[91] Zhao B., Ran J., Zhong J. (2013). Simultaneous cadmium (II)/phenol removal from water by mixed micellar-enhanced ultrafiltration using Brij 35/SDS. Asian J. Chem..

[92] Menger F.M., Portnoy C.E. (1967). Chemistry of reactions proceeding inside molecular aggregates. J. Am. Chem. Soc..

[93] Acharjee A., Rakshit A., Chowdhury S., Saha B. (2021). Micelle catalysed conversion of ‘on water’ reactions into ‘in water ’one. J. Mol. Liq..

[94] Beccia M.R., Biver T., García B., Leal J.M., Secco F., Ruiz R., Venturini M. (2012). Mechanism of Ni^2+^ and NiOH^+^ interaction with hydroxamic acids in SDS: Evaluation of the contributions to the equilibrium and rate parameters in the aqueous and micellar phase. Dalton Trans..

[95] Dupont-Leclercq L., Giroux S., Parant S., Khoudour L., Henry B., Rubini P. (2009). Complexation of Cu(II) by Original Tartaric Acid− Based Ligands in Nonionic Micellar Media: Thermodynamic Study and Applications. Langmuir.

[96] Berberich K.A., Reinsborough V.C., Shaw C.N. (2000). Kinetic and solubility studies in zwitterionic surfactant solutions. J. Solut. Chem..

[97] Fletcher P.D., Robinson B.H. (1984). Effect of organised surfactant systems on the kinetics of metal–ligand complex formation and dissociation. J. Chem. Soc. Faraday Trans. 1.

[98] Mandal H.K., Patel B.K., Dasmandal S., Mahapatra A. (2018). Kinetic investigations on the alkaline hydrolysis of tris-(1, 10-phenenthroline) Fe (II) with guar gum–surfactant interactions. J. Dispers. Sci. Technol..

[99] Singh K.V. (2020). Biomimetic membranes: Effective tool for understanding the drug biomembrane thermodynamic interactions-A review. AIP Conference Proceedings.

[100] Arslan E., Findik B.K., Aviyente V. (2020). A blind SAMPL6 challenge: Insight into the octanol-water partition coefficients of drug-like molecules via a DFT approach. J. Comput. Aided Mol. Des..

[101] Popović-Nikolić M.R., Popović G.V., Grujić M., Nikolić K.M., Agbaba D.D. (2018). A theoretical study on ionization of sartans in aqueous media and on interactions with surfactant micelles. J. Mol. Graph. Model..

[102] Oliver M., Bauza A., Frontera A., Miro M. (2016). Fluorescent lipid nanoparticles as biomembrane models for exploring emerging contaminant bioavailability supported by density functional theory calculations. Environ. Sci. Technol..

[103] John L.H., Preston G.M., Sansom M.S., Clifton L.A. (2021). Large scale model lipid membrane movement induced by a cation switch. J. Colloid Interface Sci..

[104] Pezeshkian W., Marrink S.J. (2021). Simulating realistic membrane shapes. Curr. Opin. Cell Biol..

[105] Kinnun J.J., Scott H.L., Ashkar R., Katsaras J. (2021). Biomembrane Structure and Material Properties Studied with Neutron Scattering. Front. Chem..

[106] Tsukanov A.A., Pervikov A.V., Lozhkomoev A.S. (2020). Bimetallic Ag–Cu nanoparticles interaction with lipid and lipopolysaccharide membranes. Comput. Mater. Sci..

[107] Kozma D., Simon I., Tusnady G.E. (2012). PDBTM: Protein Data Bank of transmembrane proteins after 8 years. Nucleic Acids Res..

